# The Threat of Impending Pandemics: A Proactive Approach

**DOI:** 10.7759/cureus.36723

**Published:** 2023-03-27

**Authors:** Baijayantimala Mishra, Sutapa Rath, Monalisa Mohanty, Prasanta R Mohapatra

**Affiliations:** 1 Microbiology, All India Institute of Medical Sciences, Bhubaneswar, IND; 2 Pulmonary Medicine and Critical Care, All India Institute of Medical Sciences, Bhubaneswar, IND

**Keywords:** preparedness, zoonosis, mers, sars, influenza, pandemic

## Abstract

The incessant occurrence of devastating health-related events, either on a large scale, such as pandemics, or in a local community in the form of sporadic outbreaks due to infectious agents, warrants a rapid, target-oriented, well-organized response team to combat the demonic consequences. While the world has been recovering from the clutches of the recent disastrous COVID-19 pandemic, the struggles against novel emerging and re-emerging pathogens such as monkeypox (mpox), newer evolving strains of influenza, Ebola, Zika, and the yellow fever virus continue to date. Therefore, a multisectoral, intercontinental, collaborative, interdisciplinary, and highly dedicated approach should always be implemented to achieve optimal health and avert future threats.

## Introduction and background

**Figure 1:**
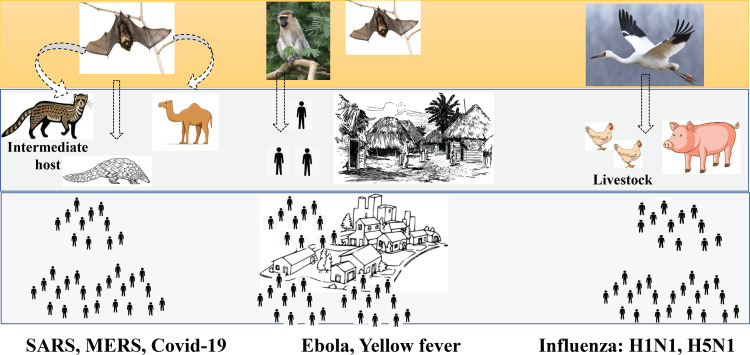
The depiction of the emergence of pandemics with an initial circulation of pathogens in wildlife followed by a spillover to humans leading to its global spread The image has been created by the authors.

Since time immemorial, the human race has been exposed to a plethora of microbes that cause deadly outbreaks, epidemics, and pandemics that continue to challenge us in ways that can cripple even the most advanced healthcare systems. Emerging and re-emerging infectious pathogens and their spread have caused serious jeopardy and led to an increased incidence of outbreaks and pandemics over the last decade. Studies document the emergence of a new human infectious disease every eight months approximately, with more than 35 emerging infectious diseases infecting humans surfacing since the 1980s [1]. In contrast, the prediction of future pandemics, their control, and outbreak investigations have been largely ignored and underfunded. Details are illustrated in Figure 1.

The past few decades have seen the emergence of many novel agents that have caused pandemics like severe acute respiratory syndrome (SARS), Middle East respiratory syndrome (MERS), Ebola, flu viruses, and the most recent severe acute respiratory syndrome coronavirus 2 (SARS-CoV-2). The emergence of these novel pandemic agents has never been predicted before their first appearance [2]. However, the patterns of their origins and spread need to be studied as part of the surveillance strategy. The occurrence of pandemics has substantially increased over time and is dominated by zoonoses (60%), of which almost 72% originate in wildlife. Thus, the threat posed by zoonoses-infectious diseases that jump from animals to humans-is rising. And the risk of a new pandemic is higher now than ever before, with the most likely scenario for the next pandemic being a new strain of influenza, like the avian influenza A (H7N9) "bird flu" virus, or a newly identified virus such as another novel coronavirus, all of which are zoonotic.

These lethal novel zoonotic agents have high pandemic potential and continue to threaten global health security [3]. Additionally, the use of a traditional model of the epidemiological triad also explains the occurrence and transmissibility of infections with pandemic potential. For example, in yellow fever, the triad of the yellow fever virus of the Flaviviridae family, the human host, and the expanding breeding places of Aedes mosquitoes due to deforestation, urbanization, and increasing air travel across continents have led to escalating opportunities for yellow fever in nonendemic countries. Similarly, in the case of Ebola virus infection, the interference in the epidemiological triad has resulted in a recent increase in the frequency of Ebola virus outbreaks in both endemic and nonendemic regions.

Major reasons for the occurrence of these pandemics include new infectious organisms crossing the species barrier from animals to humans, prolonged survival in debilitated and susceptible immunosuppressed cohorts, evolving and mutating microbes, mass population emigration, enhanced livestock production, an upsurge in wildlife trade, deforestation, expanding cities with exploding population statistics, increased travel, climate change, and escalated human-animal interactions [4]. In addition, the chain of transmission plays a pivotal role in disease transmissibility. For instance, in the SARS-CoV-2 virus and other coronaviruses like MERS, fauna closely inhabiting humans, like camels and civet bats, act as reservoirs, while the mode of transmission is via droplets or aerosol, and they more severely infect extremes of age and persons with comorbidities like diabetics. Similarly, in recent outbreaks of monkeypox in 2022, the unusual rise of cases, particularly in men having sex with men (MSM) communities predominantly in non-indigenous geographic locations, implicates the possibilities of evolution in the nature of the virus, its transmissibility route, a newer spectrum of clinical presentations, and the susceptible host.

## Review

In the last three years, the novel coronavirus disease 19 (COVID-19) pandemic has wreaked havoc globally and ravaged health and economic growth worldwide. The lingering pandemic began with the emergence of the novel virus, followed by the unfurling of numerous new variants. The World Health Organization (WHO), in collaboration with its international network of experts and researchers, has been assessing and monitoring the changes in the virus and evolution of SARS-CoV-2 since January 2020. In the late 2020s, the emergence of variants that posed an increased risk to global public health prompted the characterization of specific "variants under monitoring" (VUMs), "variants of interest" (VOI), and "variants of concern" (VOCs) for the prioritization of monitoring and research globally as well as for direct response to the pandemic. The newly emerged variants gain mutations that increase viral infectivity and transmissibility, affecting the ability of the virus to evade the protective immune response, therapeutic options, or the efficacy of currently licensed vaccines. AZ.5, C.1.2, B.1.617, B.1.630, and B.1.640 are VUMs; Lambda and Mu variants are classified as VOIs; and Alpha, Beta, Gamma, and Delta are defined as VOCs. Further, another emergent variant, Omicron, also described as VOC, shows more than thirty mutations in the spike protein, accelerating interaction with the angiotensin-converting enzyme 2 (ACE2) receptors, higher viral infectivity and transmissibility, immune resistance, and decreased lung infectivity, and hence lower pathogenicity compared with the Delta variant [5,6]. The continued evolution of the virus demands the strengthening of surveillance and sequencing abilities for a better approach to studying the extent of transmission of circulating and mutating SARS-CoV-2 variants and detecting unusual epidemiological events.

**Figure 2:**
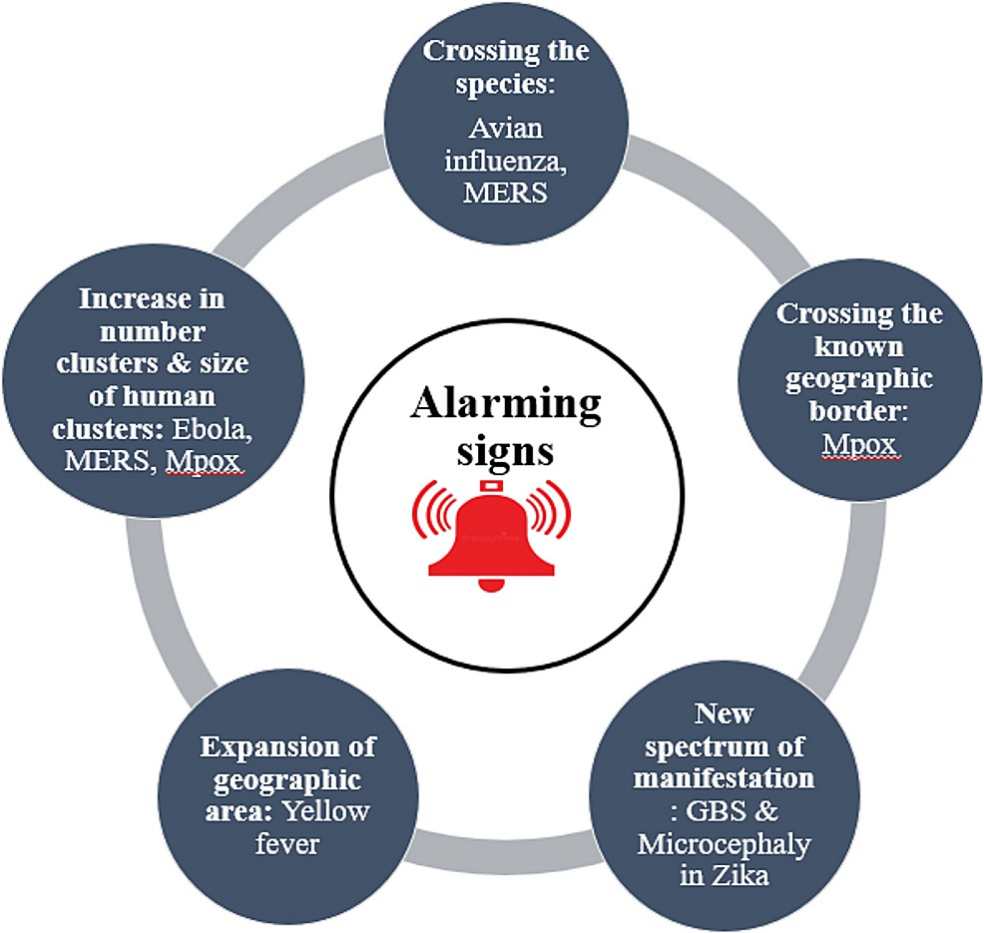
The main alarming signs of pandemics are a sudden increase in the number of cases and bigger clusters of cases beyond familiar geographical areas, the occurrence of diseases beyond known species, and the appearance of newer, unrecognized clinical manifestations

Apart from the COVID-19 pandemic, the 2009 swine flu (H1N1) influenza pandemic, Ebola, MERS, Zika virus, and monkeypox disease have been declared public health emergencies of international concern (PHEIC) [7]. In this context, with a steady rise in spill-over events from wildlife to human hosts, analyzing the major anticipated causative agents of pandemic outbreaks and identifying the triggering changes that lead to the emergence of virulent, pathogenic strains of pathogens, their susceptibility to humans needs to be assessed to tackle them in the pre-pandemic phase itself. Further, a well-articulated action plan is required to counter these health problems, along with the application of the principles of the one-health approach. Broadly, as shown in Figure 2, the impending diseases that might explode into pandemics and should never be ignored might be an existing virus with an evolving new variant: the SARS-CoV 2 virus; a new novel virus, presumably of zoonotic origin: the SARS-CoV 2 virus; a known virus in a new geographical region: the yellow fever virus; future threats due to increased human interactions and travel: monkeypox; and an existing pathogen with a newer spectrum of disease manifestations: the Zika virus.

H5N1: The Covert Influenza?

There is little doubt that the human race may be on the brink of another influenza pandemic before we can fully recover from the clutches of the COVID-19 pandemic. Human influenza is primarily transmitted by large respiratory droplets. Annual vaccination campaigns recommend standard contact precautions and antiviral therapy can help chaperone these agents. However, there is uncertainty regarding the exact modes of human-to-human transmission of avian influenza, and there is a vigilant need for additional contact precautions due to the continued evolution of the virus that might be capable of sustained human-to-human transmission. Lack of vaccination against these evolving clades and subtypes as well as higher mortality rates of greater than 50% further increase the complexity. The southern parts of China remain the hypothetical epicentre for the emergence of H5N1 clades and subclades. Despite universal vaccination of domestic poultry, H5N1 viruses are perpetuated among domestic birds prevalent in the region. Furthermore, the rising incidence of clusters of infections in Indonesia and Turkey indicates consistently sustained human-to-human transmission. Analogous circumstances that bring together all of these situations, along with obscurity in diagnosis and the unavailability of vaccines and therapeutic options, can create despoliation [8-10].

Middle East Respiratory Syndrome Coronavirus (MERS-CoV): Doomed by the Dromedaries

The MERS-CoV virus, also known as the camel flu, was first isolated from a patient with severe pneumonia in 2012 and can cause severe respiratory disease, marked by life-threatening pneumonia along with renal failure [11]. Since 2012, 27 countries have reported more than 2600 cases, with 935 known deaths due to the infection and its complications, for a case fatality rate of 36% [12]. The zoonosis, with dromedary camels as the source of infection, has been reported in the Arabian Peninsula. Outbreaks of non-sustained human-to-human transmission affecting more than 100 individuals occur, with occasional importations recurrent and outbreaks in healthcare settings in the Arabian Peninsula and the Republic of Korea [13,14]. Currently, there are numerous knowledge gaps regarding the transmission of MERS-CoV, its evolution, probable disease pathogenesis, the absence of any efficacious therapeutic options, and vaccine prospects. These sporadic and fatal MERS outbreaks, especially in hospital settings, call for a continued international collaborative approach to gain a better understanding of the virus, more effective control of animal-to-human transmission, and to prioritize research toward the development of an effective human antiviral agent and dromedary vaccine [15].

Ebola: The Massacre of Mankind

The Ebola virus, belonging to the filoviridae family, has five species: Sudan, Zaire, Tai Forest, Bundibugyo, and Reston. Bombali virus (BOMV), a novel ebolavirus that belongs to the proposed new species BOMV, has been recently detected in bats in Kenya and Sierra Leone [16]. Secondary transmission resulting from close contact between infected individuals or corpses through exposure to infectious body fluids follows initial zoonotic transmission from fruit bats. Ebola virus disease (EVD), an almost fatal zoonosis, causes fever, chills, and hemorrhagic manifestations like petechiae, ecchymoses, and internal bleeding [17,18]. Deforestation, followed by increased human-infected reservoir interaction, has been previously linked to EVD outbreaks [16]. Although there has been significant progress in research regarding the Ebola virus, various gaps remain concerning the virus ecology and ever-expanding outbreaks. The complex viral interactions of disease pathogenesis, surveillance, limited diagnostics, therapeutic options, and outbreak control dictate transmission dynamics along with the case fatality rate of an Ebola outbreak.

Monkeypox (Now “Mpox): An Unusual Turmoil Beyond Traversed Paths

Yet another zoonosis, the monkeypox disease, was characteristically Africa-limited, causing an average of a few thousand cases every year and occasional outbreaks disseminated by travel to an endemic area. The unusually rapid escalation of monkeypox cases beyond Africa as the world was recovering from the COVID-19 pandemic since May 2022 has put scientists on high alert. With more than 85,000 cases and 93 deaths reported in 110 countries as of February 15, 2023, this outbreak continues to constitute a public health emergency of international concern [19]. An alteration in virulence pattern and identification of unusual transmission modes of the virus, i.e., among men having sex with men (MSM), could be the reason for the current outbreaks indicating the emergence of strains with better transmission dynamics, though it is a rare phenomenon, especially in a large DNA virus. Atypical clinical presentations such as proctitis, urethritis, severe pain, myocarditis, and encephalitis are further perplexing [20]. The sudden upsurge of cases and its rapid global spread might have been accelerated by viral adaptation to aid human-to-human transmission of the monkeypox virus, which might have remained undiscovered for certain periods and has now redesigned itself with better specificity in the human host, as seen in SARS-CoV-2 VOCs. As scientists continue to unravel the social and epidemiological links of the mysterious outbreak, the origin, genetic, and transmission determinants; simultaneous active case detection and their isolation; contact tracing; and postexposure vaccination are being performed for impromptu containment of the disease [21].

Yellow Fever's Next Destination: Asia?

Yellow fever virus (YF), a flavivirus, and Aedes aegypti, its vector, were introduced to the Western hemisphere from Africa in the 1600s as retribution for the slave trade, resulting in major epidemics that killed thousands over 350 years in the region. Although effectively controlled by the mid-nineteenth century, the unprecedented rise of air travel in the following years, coupled with unhindered population growth and urbanization, created ideal epidemiologic ambience and socio-demographic conditions for the resurgence of the dreaded virus and its spread to newer geographical areas in infected travellers who incubated the virus. The last decade has evidenced the highest number of unvaccinated travellers being infected and exporting the virus to non-endemic nations. Although there has been no secondary transmission, the risk of importation of the virus is very high. Owing to millions of unvaccinated travellers from non-endemic countries to endemic countries, secondary transmission remains likely to occur shortly. Deprived of any immunity and prior exposure to the virus, the vastly populated Asia-Pacific region remains highly vulnerable to infection [22]. The hypothesis of cross-protection by other flavivirus exposure before YF infection exists; however, there is a possibility that the case fatality rate in those without such prior exposure could even be higher due to higher vector competence among Asian strains of Ae. aegypti, which had been reported to be much lower than its African or South American counterparts, showing a complete lack of transmission capacity to explain YF absence; but even these theories are being refuted as certain Asian vector strains are found to be more competent than their Western counterparts [23]. The combination of increased international travel from endemic to non-endemic regions, lax vaccination laws, non-immune populations, poor health infrastructure, and inadequate vaccine manufacturing capacity in at-risk nations in the face of an epidemic remains a difficult proposition for mounting an effective emergency response.

Pandemic preparedness

Pandemic preparedness focuses predominantly on research on viruses that can cause pandemics and high-priority pathogens that are most likely to threaten human health, the prediction of spillover into the human race, the occurrence of disease outside their known geographic areas, and surveillance and monitoring.

Prediction and Prevention of the Pandemic

There are several programs established for strengthening public health surveillance to issue early warning signs. The WHO Global Influenza Surveillance and Response System (GISRS) works with the prime motto of protecting people from the threat of influenza through continuous worldwide surveillance, preparedness, and response for seasonal, pandemic, and zoonotic influenza. It acts as a global platform for monitoring influenza epidemiology and disease and provides global alerts for novel and emerging influenza viruses such as H7N7, H7N9, H9N2, and other respiratory pathogens [24].

Similarly, the Global EYE strategy aims to eliminate epidemics of YF by protecting at-risk populations, preventing international spread, and ensuring rapid outbreak containment [25]. The "stop monkeypox outbreak" mission of the WHO focuses on three aspects: minimizing zoonotic transmission, interrupting human-to-human transmission for the population at greater risk of mpox exposure, and protecting the vulnerable group at risk of developing the severe disease [20].

Targeted Genomic Surveillance in Hotspots

Regions where human movement occurs around the horizon of abundant wildlife biodiversity intermingled with a vast array of microbial biodiversity, are designated as hotspots. Targeted surveillance in hotspots is imminent in the detection of emerging infectious diseases and their control [2].

**Figure 3:**
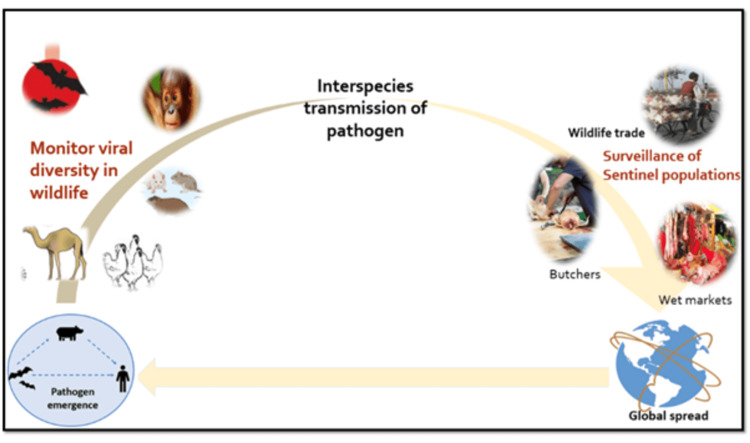
The roadmap to a strategic approach to genomic surveillance includes: the identification of hotspots of emerging infections on a global level; periodic and systematic scanning for zoonotic spillovers, especially among sentinel populations like wet market workers, butchers, vets, and susceptible animals; surveillance of known pathogens with pandemic potential The image has been created by the authors.

The PREDICT component of the Emerging Pandemic Threats programme, under the United States Agency for International Development (USAID), has developed predictive modelling approaches to identify the hotspot regions where experts from different specialities like epidemiology, virology, genetics, informatics, and veterinary medicine work in active collaboration in hotspots in 20 developing countries, focusing on surveillance and building of diagnostic capacities at human-animal interfaces where cross-species transmission is common [2] as illustrated in Figure 3.

The following aspects are covered via surveillance. 

Surveillance of animals (wild animals, domestic animals, migratory birds, and poultry); surveillance of the sentinel population (wet market workers, butchers, vets, and hunters) for known viruses of pandemic potential; surveillance of countries with inadequate sanitation and hygiene, the lack of infrastructure to deliver an intervention, and limited resources for control of zoonoses and vector-borne disease.

Rapid Detection of the Pandemic

As we proceed towards a real-time PCR (polymerase chain reaction)-based pathogen detection system, the establishment of genomic-based diagnostic laboratories that can detect microbes in the field itself with a lower turnaround time of a few hours and can perform sequencing directly from the sample is the need of the hour. Hence, a portable "lab-in-a-suitcase" sequencing platform rather than a bench-top instrument is a pressing necessity [26]. The development of newer diagnostics should focus on meeting the "ASSURED" (Affordable, Sensitive, Specific, User-Friendly, Rapid/Robust, Equipment-Free, and Deliverable) criteria of the WHO [27].

Tracking of pathogen strains to comprehend the emergence of various variants like VOIs and VOCs is pertinent for the control of the pandemics.

Regarding the role of digital technology in pandemic preparedness and response, "digital disease detection" is the current approach to disease surveillance synonymous with digital epidemiology. At least 50 digital disease detection systems are currently in place that retrieve information from a variety of sources, including newswires, digital media, official reports, and crowdsourcing; translate, process, and analyze their trends; and then disseminate this information to the community via websites, emails, media, and mobile alerts [26].

Controlling the Pandemics

The close interplay and balance between animals, humans, and their environments call for a one-health collaborative approach to controlling pandemics. Therefore, preparing for future pandemics needs special emphasis as well as national and international level funding for multidisciplinary collaboration of different scientific streams under the one-health strategy. Such a multi-level combination of research approaches will accelerate pandemic control and prevention with knowledge of pathogen origin and adaptation, strengthen infrastructure and networks for diagnostics, and enhance vaccine and therapeutic research capabilities, allowing the mitigation of emerging pandemics.

## Conclusions

Incessant land use changes, exploding population statistics, continuous genetic evolution at the pathogen level, and extravagant human-flora-fauna interaction in the ecological niches induce unavoidable zoonotic spills, which require a hawk-eye vigil to seize them at an early phase to prevent socio-economic and health chaos. Thus, a highly dedicated one-health approach that is collaborative, interdisciplinary, multi-sectoral, and implemented across international borders is the ultimate need of the hour for the prevention of future threats. A workable multi-sectoral accountability framework and program reforms are also needed for the prevention of futuristic threats, in addition to carefully following the steps of an outbreak or pandemic investigation.
